# A Replication-Competent Retroviral Vector Expressing the HERV-W Envelope Glycoprotein is a Potential Tool for Cancer Gene Therapy

**DOI:** 10.4014/jmb.2309.09022

**Published:** 2023-12-18

**Authors:** Byoung Kwon Kang, Yong-Tae Jung

**Affiliations:** Department of Microbiology, Dankook University, Cheonan 31116, Republic of Korea

**Keywords:** Cancer gene therapy, FMG, HERV-W *env*, RCR vector, s-RCR vector, syncytium

## Abstract

The fusogenic membrane glycoprotein (FMG) derived from the human endogenous retrovirus-W (HERV-W) exhibits fusogenic properties, making it a promising candidate for cancer gene therapy. When cells are transfected with HERV-W FMG, they can fuse with neighboring cells expressing the receptor, resulting in the formation of syncytia. These syncytia eventually undergo cell death within a few days. In addition, it has been observed that an HERV-W *env* mutant, which is truncated after amino acid 483, displays increased fusogenicity compared to the wild-type HERV-W *env*. In this study, we observed syncytium formation upon transfection of HeLa and TE671 human cancer cells with plasmids containing the HERV-W 483 gene. To explore the potential of a semi-replication-competent retroviral (s-RCR) vector encoding HERV-W 483 for FMG-mediated cancer gene therapy, we developed two replication-defective retroviral vectors: a *gag-pol* vector encoding HERV-W 483 (MoMLV-HERV-W 483) and an *env* vector encoding VSV-G (pCLXSN-VSV-G-EGFP). When MoMLV-HERV-W 483 and pCLXSN-VSV-G-EGFP were co-transfected into HEK293T cells to produce the s-RCR vector, gradual syncytium formation was observed. However, the titers of the s-RCR virus remained consistently low. To enhance gene transfer efficiency, we constructed an RCR vector encoding HERV-W 483 (MoMLV-10A1-HERV-W 483), which demonstrated replication ability in HEK293T cells. Infection of A549 and HT1080 human cancer cell lines with this RCR vector induced syncytium formation and subsequent cell death. Consequently, both the s-RCR vector and RCR encoding HERV-W 483 hold promise as valuable tools for cancer gene therapy.

## Introduction

Human endogenous retroviruses (HERVs) make up about 8% of the human genome. HERV proviruses typically have two long terminal repeats (LTRs) flanking the *gag*, *pro*, *pol*, and *env* genes. However, most HERVs are non-functional due to genetic mutations. The HERV-W *env* (syncytin-1) gene, located at the HERV-W provirus locus 7q21.2 (ERVWE1), plays a critical role in normal placental development [1,2]. This gene encodes a glycoprotein called syncytin-1, which has a molecular weight of 73 kDa and consists of 538 amino acids. Syncytin-1 is cleaved into a 50 kDa surface unit (SU) and a 24 kDa transmembrane unit (TM). The protein is highly fusogenic and is naturally expressed in the syncytiotrophoblast layer of the placenta.

The HERV-W Env protein interacts with the human sodium-dependent neutral amino acid transporter 2 (hASCT2; gene name, SLC1A5), which is a receptor for type D mammalian retroviruses [3]. Previous studies have shown that HERV-W *env* can also use the human sodium-dependent neutral amino acid transporter 1 (hASCT1; gene name, SLC1A4) as a receptor, even though the two receptors share only 57% similarity [4-6]. hASCT2 is a receptor for various retroviruses, including baboon endogenous virus (BaEV), feline endogenous virus (RD114), simian retroviruses (SRV-1, SRV-2), and spleen necrosis virus (SNV). hASCT2 is widely expressed in different human cell types, and HEK293T cells express low levels of the two HERV-W *env* receptors [3]. Interestingly, recent studies have reported high expression of hASCT2 in various cancer types [7,8]. Therefore, targeting HERV-W *env* through hASCT2 could offer a promising therapeutic approach for cancer treatment.

Recent studies suggest that HERV-W *env* plays a significant role in the development of autoimmune arthritis, diabetes, multiple sclerosis (MS), and schizophrenia [9-12]. Additionally, HERV-W *env* has been shown to induce syncytia formation, which is the fusion of HERV-W *env*-expressing cells with approximately 40 neighboring cells expressing the receptor [13]. This phenomenon, known as the strong bystander effect, suggests that HERV-W *env* could be a promising candidate for cancer gene therapy due to its potential to induce tumor cell death. Another advantage of employing HERV-W *env* in cancer gene therapy is its ability to elicit a mild immune response, as it is a human antigen.

Type C and D retroviral Env proteins have an R peptide, a 16-amino acid cytoplasmic tail of the transmembrane protein that inhibits fusion activity. Mutations in the R peptide can alter envelope fusion, as seen in Moloney murine leukemia virus (MoMLV), amphotropic murine leukemia virus (A-MuLV), Gibbon-ape leukemia virus (GALV), and porcine endogenous retrovirus (PERV) [14-18]. Although HERV-W *env* does not contain an R peptide-like sequence and a cleavage site to remove 16 amino acids from the C-terminus, HERV-W *env* mutants truncated after amino acid 480 are known to form larger syncytia than the wild ones [19,20]. This suggests that the cytoplasmic tail of HERV-W *env* likely contains a sequence that governs its fusogenic activity. Supporting these results, Kitao *et al*. reported that HERV-W retains a syncytin-1 post-transcriptional regulatory element (SPRE) to promote efficient gene expression [21]. The SPRE is in the 3' untranslated region (3'UTR) of the *env* gene and is essential for intron-dependent efficient expression of HERV-W.

Recombinant MoMLV-based retroviral vectors, such as RCR vectors, have shown significant efficacy in reducing tumors in a variety of cancer models [22-27]. One specific RCR vector incorporating an amphotropic 10A1 MLV *env* and yeast cytosine deaminase (CD) at the *env*-3'-untranslated region (3'-UTR) demonstrated notable effectiveness in killing tumor cells upon administration of its corresponding prodrug. Additionally, not only have GALV-derived RCR vectors been developed, but so too has an s-RCR vector which is designed to improve transgene insertion capacity [28-30]. This s-RCR vector system consists of two replication-defective vectors, *gag-pol* and *env*, and is designed to produce infectious viral particles in cells co-expressing both vectors. In addition, it was reported that lentiviral vectors can be pseudotyped with the HERV-W *env*, but the HERV-W *env* cannot be incorporated into MLV particles [31]. Therefore, HERV-W *env* in the MLV vector does not interfere with MLV *env*.

In this study, a truncated mutant comprising 483 residues was employed to generate an oncolytic RCR vector and an s-RCR vector. HEK293T cells were transfected with these vectors and their effects were examined. Both RCR and s-RCR vectors elicited syncytium formation within the transfected cells. Subsequently, human cancer cell lines were infected with supernatants derived from RCR vector-transfected HEK293T cells, resulting in viral transmission and cell death due to HERV-W 483-induced toxicity. These findings provide evidence that the expression of HERV-W 483 in tumor cells induces cell-cell fusion and eventual cell death.

## Materials and Methods

### Cell Lines

The HEK293T human embryonic kidney cells (ATCC CRL-11268), A549 human lung adenocarcinoma cells (ATCC CCL-185), HeLa human cervix carcinoma cells (ATCC CCL2), HT1080 human fibrosarcoma cells (ATCC CCL-121), and TE671 human rhabdomyosarcoma cells (ATCC CRL-8805) were maintained in Dulbecco’s modified Eagle medium (DMEM) supplemented with 10% fetal calf serum (FCS), 100 U/ml penicillin, and 100 μg/ml streptomycin.

### Construction of HERV-W 483 Mutant

HERV-W *env* and HERV-W 483 mutant were obtained from the pcDNA3.1-ERVW-1 (GenScript, USA) plasmid through PCR amplification. The primers used were as follows: HERV-W *env* F: 5'-CCATGGCCCTCCCTTATCAT- 3' (NcoI restriction site is underlined), HERV-W *env* R: 5'-GCGGCCGCCTAACTGCTTCCTGCTGA-3' (NotI restriction site is underlined), HERV-W 483 F: 5'-CCATGGGCCACCATGGCCCTCCCTCA-T-3' (NcoI restriction site is underlined), and HERV-W 483 R: 5'-GCGGCCGCCTATACAGC-TTCGATTCTGGA-3' (NotI restriction site is underlined). The PCR products were ligated into the pGEM-T Easy Vector System (Promega, USA); afterwards, the pGEM-T-HERV-W *env* or the pGEM-T- HERV-W 483 was digested with the restriction enzyme EcoRI. The digested fragment was ligated into pCMV-VSV-G (Addgene #8454, USA) to generate pCMVHERV- W *env* or pCMV-HERV-W 483. To construct pCMV-HERV-W-SPRE, we first inserted the synthetic 200 bp of SPRE into pcDNA3.1(-) using the NheI and NotI sites present in the synthetic SPRE to generate pcDNA3.1(-) SPRE. Then, the NheI-NheI fragment of pcDNA3.1-ERVW-1 was inserted into the NheI site of pcDNA3.1(-) SPRE to generate pcDNA3.1(-) HERV-W SPRE. Finally, the BamHI-BamHI fragment of pcDNA3.1(-) HERV-W SPRE was inserted into pCMV-VSVG to complete pCMV-HERV-W-SPRE. To detect expression of HERV-W 483, three hemagglutinin (HA) epitope tags were added to the C-terminus of HERV-W 483 (pcDNA3 intron HERV-W 483 HA3). pcDNA3 intron HERV-W 483 HA3 was generated by introducing the PCR product of HERV-W 483 into the pcDNA3-mCAT-HA vector using the BamHI and XhoI sites present in the primers. Primary antibodies (anti-HA antibody, 1:1,000) (SantaCruz, USA) and secondary antibodies (antimouse IgG HRP, 1:5,000) (AbClon, Republic of Korea) were used for western blotting. To investigate the differential fusogenic activity of pCMV-HERV-W, pCMV-HERV-W-SPRE, and pCMV- HERV-W 483, HEK293T cells were transfected with HERV-W expression plasmids. Human cancer cell lines HeLa and TE671 were also transfected with pCMV-HERV-W 483. At 48 h post-transfection, syncytium formation was observed using light microscopy. To test the cytotoxic effect of various HERV-W-expressing plasmids on HEK293T cells, relative cell viabilities was determined using the MTT assay kit (Intron, Republic of Korea) according to the manufacturer’s protocol.

### Production of HERV-W 483 Pseudotyped HIV-1 Particles

To determine whether the HERV-W 483 mutant can serve as a functional membrane glycoprotein for pseudotyping HIV-1 virions, HEK293T cell lines were co-transfected with 1 μg of psPAX2 (Addgene #12260), 1 μg of pLenti-CMV-GFP-Puro (Addgene #17448), and one of the HERV-W envelope protein expression plasmids (pCMV-HERV-W, pCMV-HERV-W-SPRE, and pCMV-HERV-W 483, 1 μg) using the Calphos Mammalian Transfection Kit (TaKaRa, Japan). Virus-containing supernatants were harvested 48 h after transfection and HEK293T cells were infected with virus in the presence of polybrene (8 μg/mL). To determine the viral titers, GFP-positive cells were analyzed using a FACSCalibur flow cytometer (Becton, Dickinson and Company, USA).

### Construction of RCR Vector Encoding HERV-W 483 Mutant

To investigate whether hyperfusogenic HERV-W 483 has antitumor effects, we generated MoMLV-10A1-HERV-W 483 by replacing EGFP in the previously constructed MoMLV-10A1-EGFP RCR vector with HERV-W 483 [16]. First, to construct IRES-HERV-W 483, the XhoI-NcoI PCR product of IRES from pIRES2-EGFP (Clontech, USA) was ligated with the NcoI/NotI fragment containing HERV-W 483 from pGEM-T- HERV-W 483. The IRES-HERV-W 483 fragment was then transferred into XhoI/NotI-digested pLPCX (Clontech) to create pLPCX-IRES-HERV-W 483. Finally, the NheI/NheI fragment containing IRES-HERV-W 483 was ligated into NheI-digested MoMLV-10A1-EGFP to generate MoMLV-10A1-HERV-W 483. To investigate whether MoMLV-10A1-HERV-W 483 is capable of replicating in HEK293T cells, HEK293T cells were transfected with RCR vector and was passaged for 15 days. For virus production, HEK293T cells were transiently co-transfected with MoMLV-10A1-HERV-W 483 and pCLXSN-EGFP using the CalPhos Mammalian Transfection Kit. Virus-containing supernatants were harvested 72 h after transfection and were used to infect A549 and HT1080 cells at an MOI of 1.

### Construction of s-RCR Vector Encoding HERV-W 483 Mutant

To construct the s-RCR vector, MoMLV-10A1-HERV-W 483 vector was digested using EcoRI and then re-ligated to remove the 10A1 *env* gene (MoMLV-HERV-W 483). To generate the replication-defective vector-expressing *env* gene, VSV-G was first amplified from the pCMV-VSV-G plasmid through PCR and then ligated into the pCLXSN-IRES-EGFP vector previously digested with EcoRI/NotI to generate pCLXSN-VSV-G. Secondly, the IRES-EGFP fragment was transferred into the NheI/NheI-digested pCLXSN-VSV-G to create pCLXSN-VSV-G-EGFP. For s-RCR vector production, 1 μg of MoMLV-HERV-W 483 and 1 μg of pCLXSN-VSV-G-EGFP were co-transfected into HEK293T cells using the CalPhos Mammalian Transfection Kit. Viral titers were calculated by counting syncytia-positive cell numbers (syncytial-forming units; sfu/ml).

## Results

### HERV-W 483 Induced Syncytium Formation

**Fig. 1:**
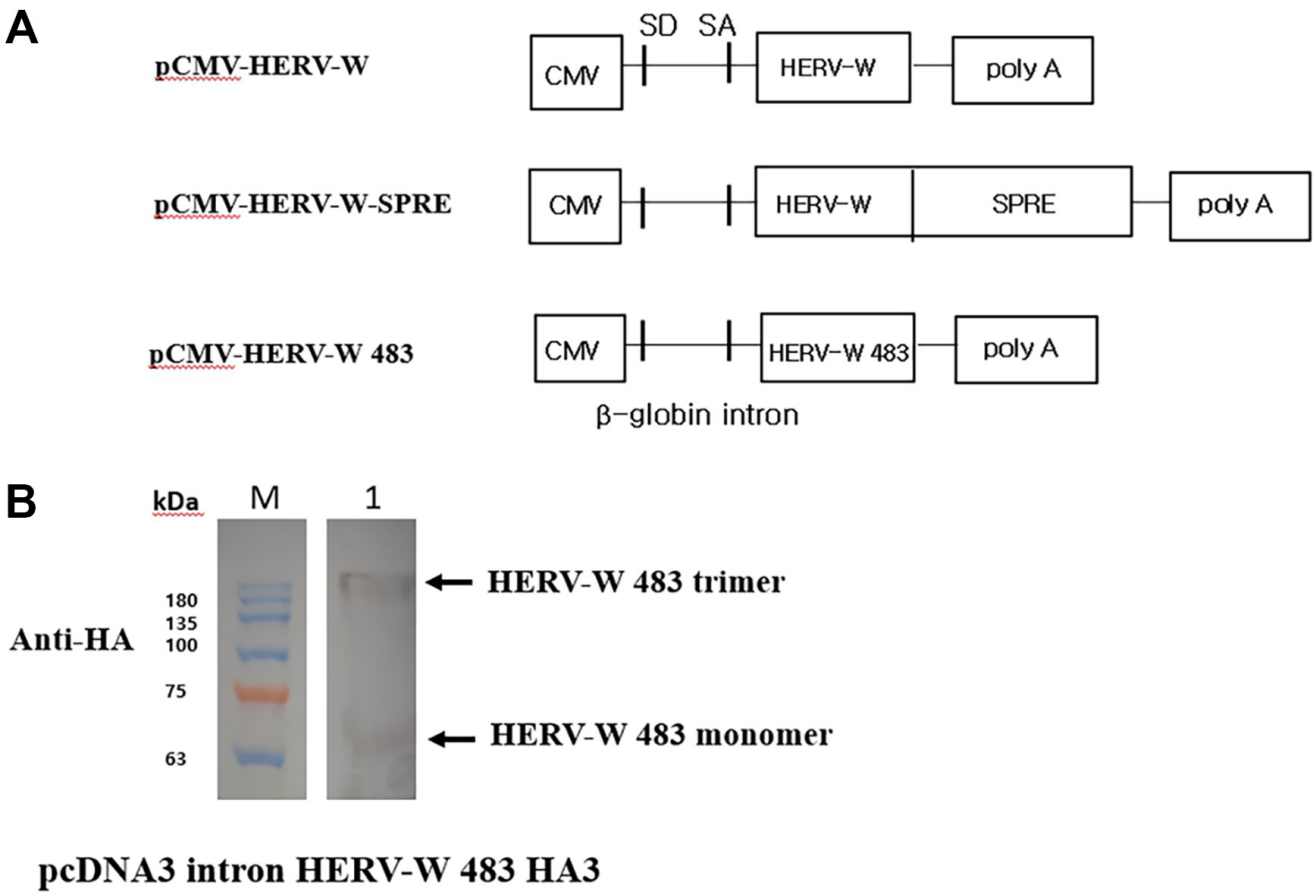
Schematic representation of HERV-W *env* expression plasmids and HERV-W *env* expression in transfected HEK293T cells. (**A**) The location of MLV splice donor(SD), splice acceptor(SA), and β-globin intron site are indicated. The syncytin-1 post-transcriptional regulatory element (SPRE) encompassing the 100 bp of ORF and 100 bp of the 3’UTR of HERV-W were inserted into pCMV-HERV-W to generate pCMV-HERV-W-SPRE. HERV-W mutant truncated after amino acid 483 was cloned into plasmid pCMV-VSV-G to construct pCMV-HERV-W 483. (**B**) The expression of HERV-W 483 was analyzed using western blotting. HEK293T cells were transfected with pcDNA3 intron HERV-W 483 HA3, and immunoblot of cell lysates were probed with an anti-HA antibody.

**Fig. 2:**
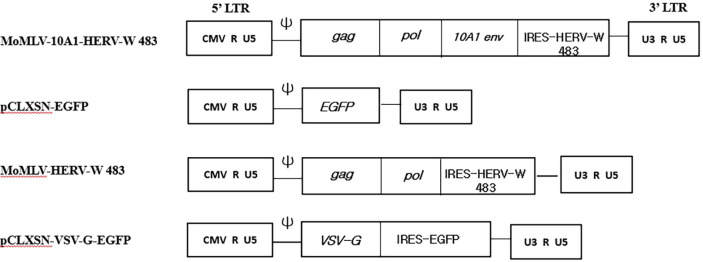
Plasmid structure of the replication-competent retroviral (RCR) and semi-replication-competent retroviral (s-RCR) vectors. The RCR and s-RCR vectors are derived from the wild-type Moloney Murine Leukemia Virus (MoMLV). The MoMLV-10A1-HERV-W 483 RCR vector was created by replacement of IRES-EGFP cassette of MoMLV- 10A1-EGFP RCR vector with IRES-HERV-W 483. To generate s-RCR vector, two separate packageable vectors (MoMLVHERV- W 483 and pCLXSN-VSV-G-EGFP) were constructed. MoMLV-HERV-W 483 is a *gag-pol* vector containing HERV-W 483, and pCLXSN-VSV-G-EGFP is an *env* vector constructed by inserting VSV-G into the pCLXSN backbone. Ψ: packaging signal, LTR: long terminal repeat.

**Fig. 3:**
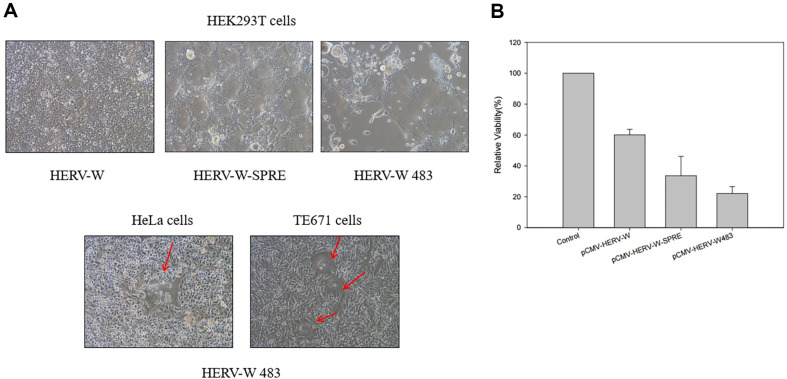
Analysis of HERV-W 483-mediated syncytium formation and cytotoxicity. (**A**) HEK293T cells were transiently transfected with plasmids encoding HERV-W, HERV-W-SPRE, and HERV-W 483. In addition, human cancer cell lines HeLa and TE671 were transfected with pCMV-HERV-W 483. Cells were examined for syncytium formation using light microscopy at 200x magnification. Arrows indicate syncytia. (**B**) To investigate the cytotoxic activities of HERV-W, HEK293T cells were transfected by pCMV-HERV-W, pCMV-HERV-W-SPRE, and pCMV-HERV-W 483. Cell viabilities were determined using the MTT assay 3 days after transfection. Results are presented as the means ± SD of the three independent experiments performed in triplicate.

To compare the fusogenic activities of HERV-W *env*, HERV-W *env* SPRE, and HERV-W 483 mutants, we constructed HERV-W envelope protein expression plasmids (Fig. 1A). Expression of HERV-W 483 was confirmed in HEK293T cells transfected with the pcDNA3 intronic HERV-W 483 HA3 vector, where the low-molecular-mass band (65 kDa) corresponds to the monomeric HERV-W 483 precursor (Fig. 1B). To rapidly induce syncytia and maintain genome integrity, semi-replication-competent retroviral vectors encoding either VSV-G or HERV-W 483 were constructed. The *gag-pol* and *env* vectors, which constitute the s-RCR vector, contain HERV-W 483 and VSV-G, respectively (Fig. 2). Previous studies reported that the cytoplasmic tail of HERV-W *env* inhibits its fusogenicity [19, 20]. Consistent with these findings, HEK293T cells transfected with HERV-W 483 induced syncytium formation more efficiently than HEK293T cells transfected with HERV-W *env* or HERV-W *env* SPRE (Fig. 3A). Interestingly, HEK293T cells did not form syncytia when transfected with this HERV-W *env* expression plasmid without SPRE. However, these results contradict previous findings that HERV-W *env* is a highly fusogenic membrane glycoprotein [3]. Blond *et al*. used phCMV-ENVpH74 as a HERV-W envelope expression vector, which contains 138 bp of sequence downstream of the HERV-W *env* stop codon. These additional 138 base pairs appear to be involved in SPRE-like, post-transcriptional regulation.

To determine whether HERV-W 483 could induce syncytium formation in human cancer cell lines, HeLa and TE671 cells were transfected with plasmids encoding HERV-W 483. As shown in Fig. 3A, syncytium formation was observed in the transfected human cancer cell lines.

### Characterization of HERV-W 483 Envelope Protein

To quantify the cytotoxic activities of the HERV-W *env*, HEK293T cells were transiently transfected by HERV-W *env*, HERV-W *env* SPRE, and the HERV-W 483 mutant. Cell viabilities were measured by MTT assay 3 days after transfection (Fig. 3B). The results showed that HERV-W *env* SPRE killed ~60% of the cells, while HERV-W 483 showed even stronger killing effects at ~80% loss of cell viability.

**Fig. 4:**
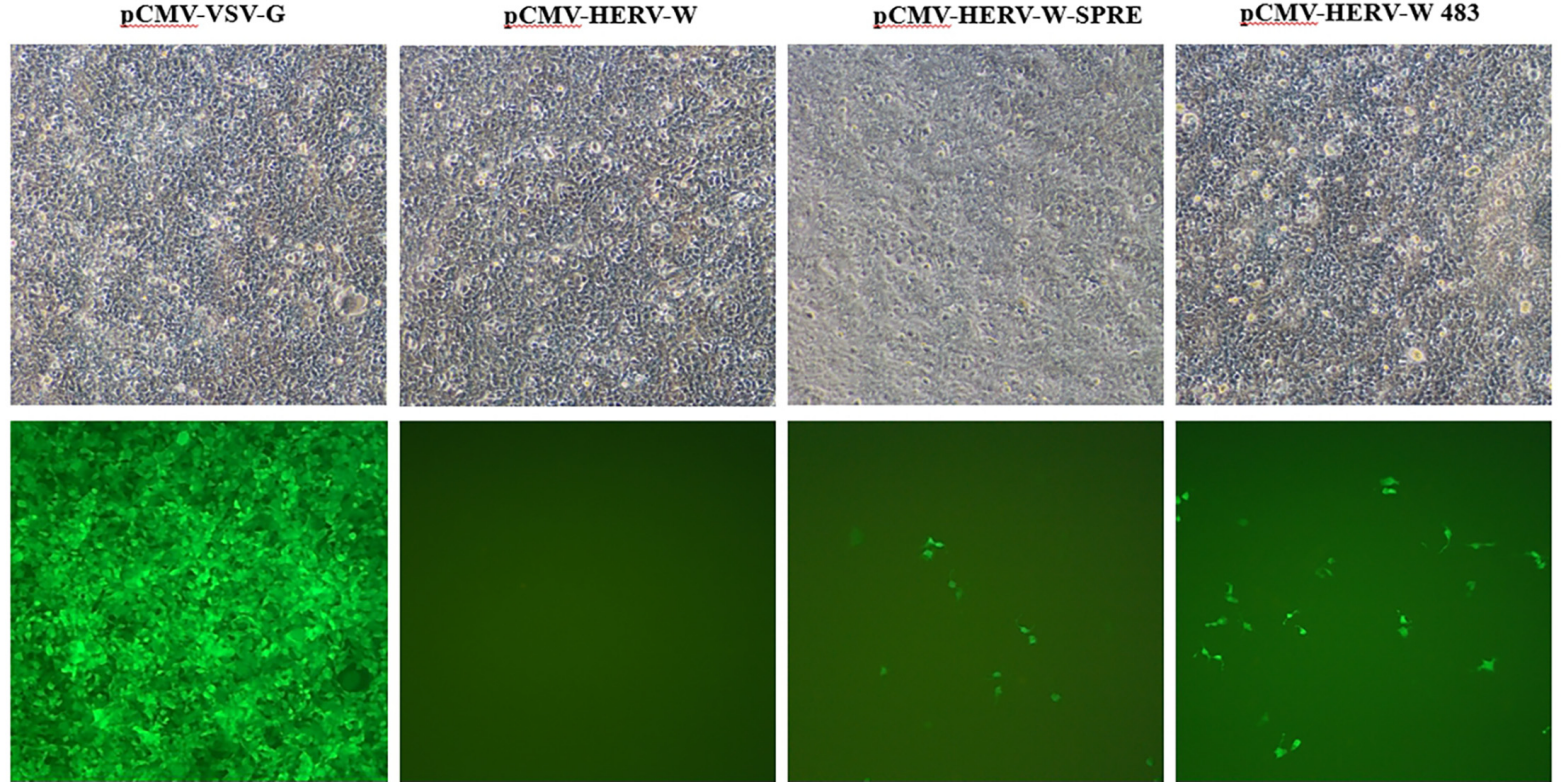
Truncation in the carboxyl-terminal end of HERV-W *env* enhances infectivity of HIV-1/HERV-W pseudotyped virus. Lentiviral particles were produced through the co-transfection of HEK293T cells with the following plasmids: pCMV-VSV-G, a vector expressing the HERV envelope (pCMV-HERV-W, pCMV-HERV-W-SPRE, and pCMVHERV- W 483), pLenti-CMV-GFP-Puro, and psPAX2. pCMV-VSV-G was used as positive control vector. Lentiviral particles were obtained 48 h after transfection before inducing extensive syncytium formation. The infectivity of lentiviral particles in HEK293T cells was quantified by measuring % GFP positive cells by flow cytometer.

To confirm whether HERV-W 483 can be incorporated into lentiviral vectors, HERV-W 483 pseudotyped HIV-1 was generated (Fig. S1). As expected, the lentiviral vectors pseudotyped with HERV-W 483 (3 × 10^4^ transduction units(TU)/ml) were more infectious than wild-type HERV-W-SPRE (1 × 10^4^ TU/ml in HEK293T cells (Fig. 4 and S2). The viral titers from control vector pCMV-VSV-G were 7 × 10^5^ TU/ml. In the subsequent experiment, an intron-containing vector was employed to explore the packaging potential of HERV-W 483 into a pseudotyped retroviral vector. As illustrated in Fig. S3, the inclusion of introns in the vectors led to a significant enhancement in pseudotyped virus production compared to intron-free vectors.

### Characterization of s-RCR Vector Encoding HERV-W 483

**Fig. 5:**
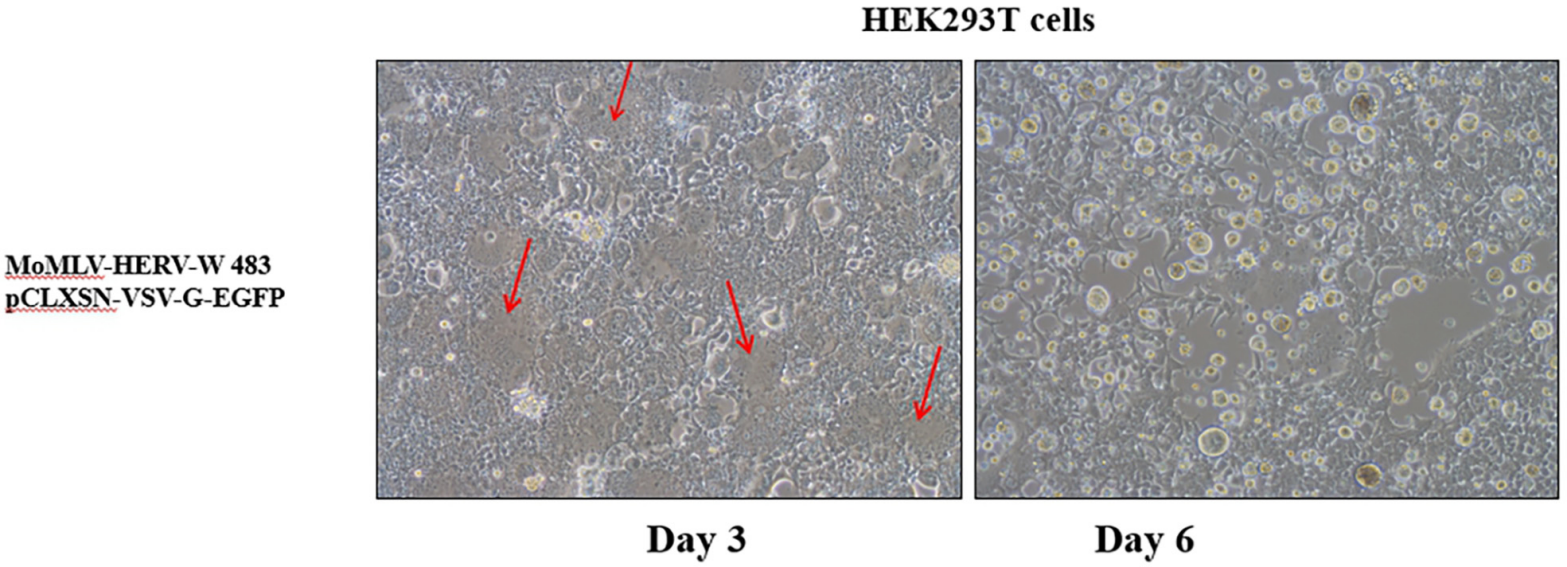
Production of semi-replication-competent retroviral (s-RCR) virus. HEK293T cells were co-transfected with a *gag-pol* vector (MoMLV-HERV-W 483) and an *env* vector (pCLXSN-VSV-G-EGFP), and syncytium formation was observed using light microscopy at 200x magnification at days 3 and 6. Arrows indicate syncytia.

When MoMLV-HERV-W 483 and pCLXSN-VSV-G-EGFP were co-transfected into HEK293T cells, we observed that they progressively induced syncytium formation in the transfected cells (Fig. 5). Since HERV-W 483 cannot be incorporated into MLV particles, the spread of the s-RCR vector is caused by VSV-G Env protein. However, titers of the s-RCR vector collected from HEK293T cells transfected with MoMLV-HERV-W 483 and pCLXSN-VSV-G-EGFP were low (<10^2^ sfu/ml). These results suggest that direct toxicity of the two FMGs (HERV-W 483 and VSV-G) reduces virus production. Although the titer of the s-RCR vector was low, VSV-G will act as both a viral envelope for efficient delivery of HERV-W 483 and a cytotoxic protein.

### Characterization of RCR Vector Encoding HERV-W 483

**Fig. 6:**
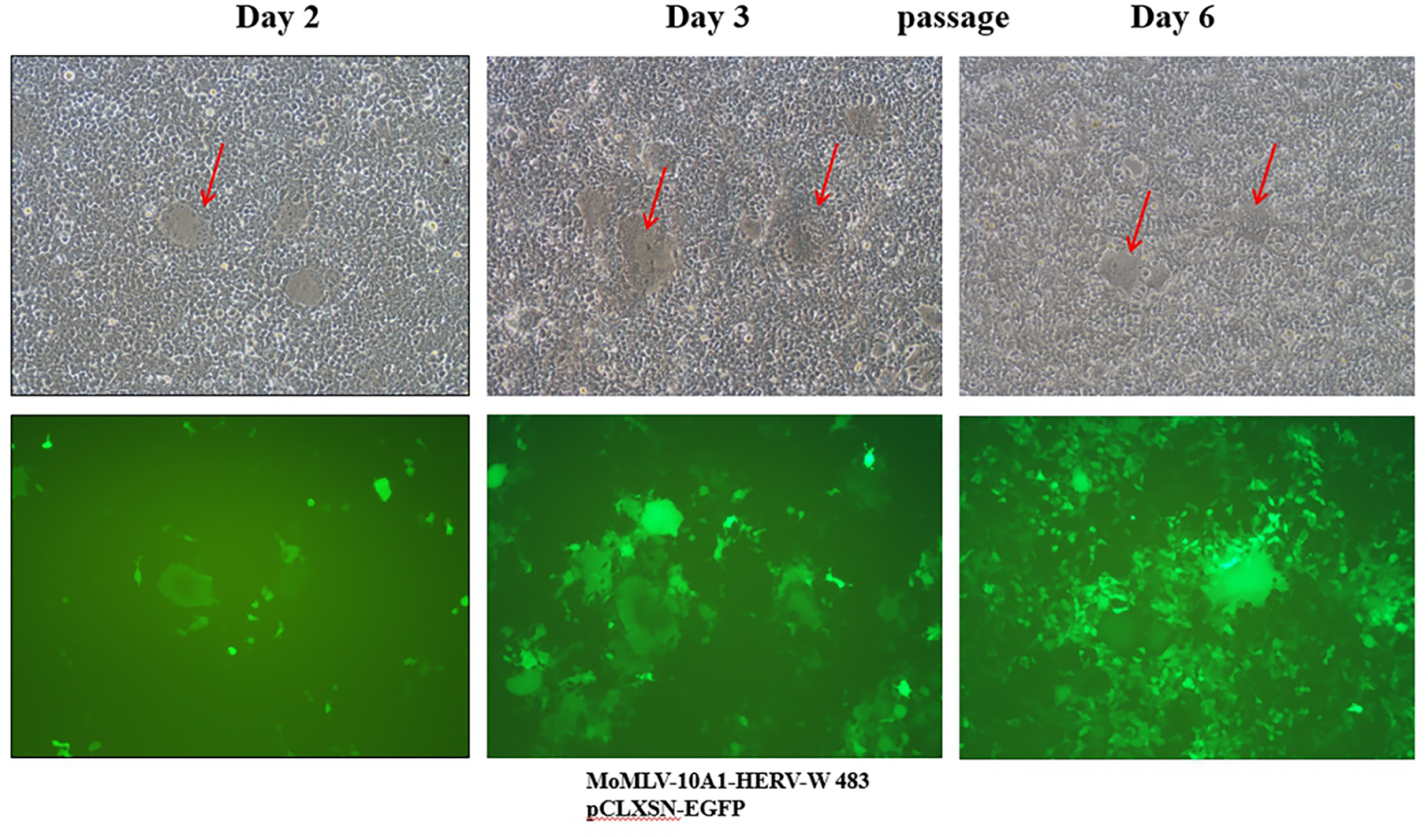
Syncytium formation by the replication-competent retroviral (RCR) vector encoding HERV-W 483. To produce RCR viruses, HEK293T cells were co-transfected with MoMLV-10A1-HERV-W 483 and pCLXSN-EGFP plasmids. Transfected HEK293T cells were subcultured 3 days after transfection, and syncytium formation was observed at 100x magnification using light microscopy (top panel) or fluorescence microscopy (bottom panel). Arrows indicate syncytia.

To determine whether the RCR vector encoding HERV-W 483 induces syncytium formation, the NheI/NheI fragment containing IRES-HERV-W 483 was subcloned into MoMLV-10A1-EGFP RCR vector (MoMLV-10A1-HERV-W 483). To clearly monitor syncytium formation, HEK293T cells were co-transfected with pCLXSN-EGFP and MoMLV-10A1-HERV-W 483. At 48 h post-transfection, syncytium formation occurred in the MoMLV-10A1-HERV-W 483-transfected cells. As shown in Fig. 6, spots of homogeneously faint green cytoplasm indicate fusion between HERV-W 483-expressing cells and their neighboring cells. When HEK293T cells transfected with pCLXSN-EGFP and MoMLV-10A1-HERV-W 483 were passaged on day 3, the proportion of EGFP-positive cells increased and syncytia were still detected after cell passaging. These results suggest that MoMLV-10A1-HERV-W 483 is a replication-competent vector that allows helper-dependent propagation of transgenes, although RCR vectors containing inserts larger than 1.3 kb are known to be unstable [32].

### RCR Vector Encoding HERV-W 483 Induced Syncytium Formation in Cancer Cells

**Fig. 7:**
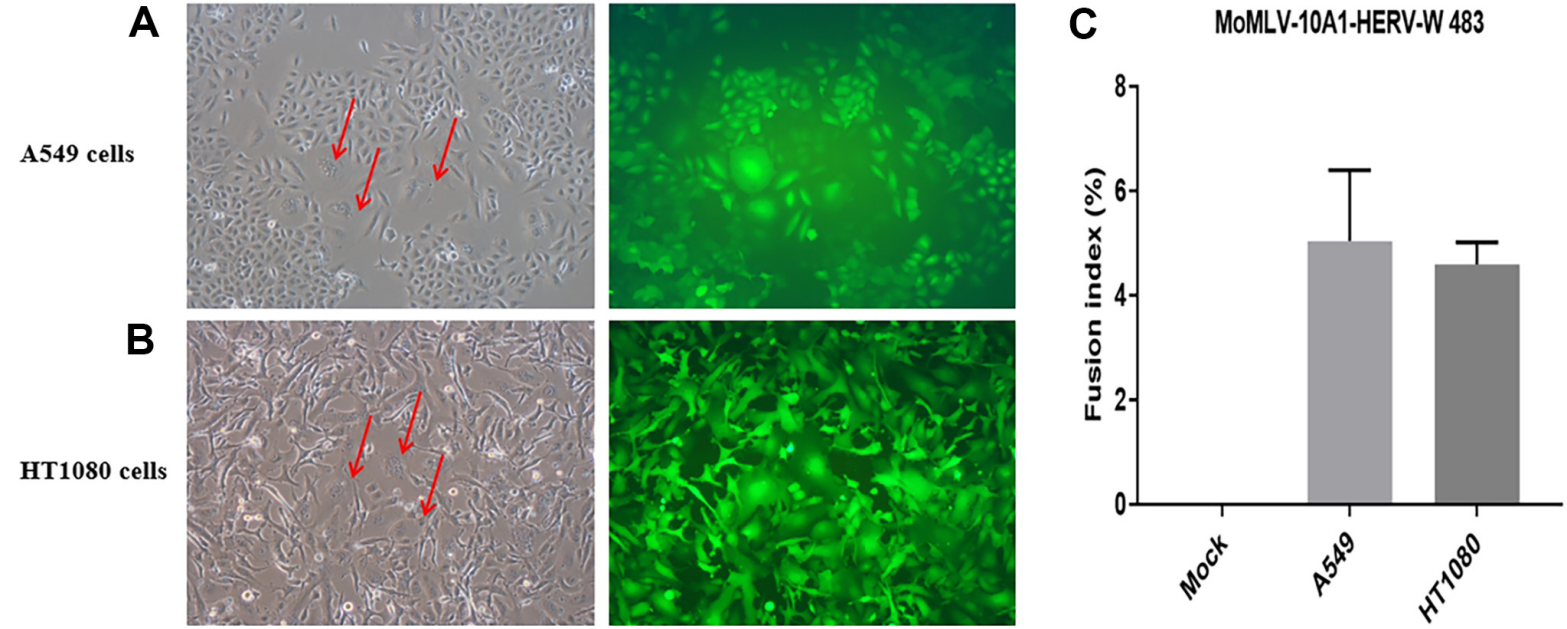
HERV-W 483-expressing RCR virus kills cancer cells by inducing syncytia formation. To generate the HERV-W 483-expressing RCR virus, HEK293T cells were transiently transfected with MoMLV-10A1-HERV-W 483 and pCLXSN-EGFP. Subsequently, supernatants collected from transfected HEK293T cells were inoculated into A549 (**A**) and HT1080 (**B**) cells, and syncytium formation was detected after 72 h. (**C**) Quantitation of syncytia formation in A549 and HT1080 cell lines infected with HERV-W 483-expressing RCR virus. The fusion index is defined as *(N-S)/T* × 100, where *N* is the number of nuclei in the syncytia, S is the number of syncytia, and T is the total number of nuclei counted. Data are the means ± SD from four fields. Magnification, 200x. Arrows indicate syncytia.

A previous study reported that HERV-W *env* is a very potent and promising tool for the treatment of human lung tumors [13]. In this study, we investigated the cytotoxic activity of HERV-W 483 on A549 and HT1080 cells. When A549 and HT1080 cells were infected with the supernatants (2 × 10^3^ sfu/ml) collected from MoMLV-10A1-HERV-W 483 + pCLXSN-EGFP co-transfected HEK293T cells, syncytium formation was observed at 3 days post-infection (Fig. 7). However, some syncytia did not show fluorescence. This is because the pCLXSN-EGFP defective retroviral vector is propagated more rapidly than the wild-type MoMLV-10A1-HERV-W 483 helper vector. Therefore, syncytia showing fluorescence means that MoMLV-10A1-HERV-W 483 and pCLXSN-EGFP co-expressed in single cell. When quantitation of cell-cell fusion induced by HERV-W 483 was performed in A549 and HT1080 cells, HERV-W 483 showed a slightly higher fusion index in A549 than in HT1080 cells (Fig. 7C).

## Discussion

HERV-W *env*-expressing cells can fuse with many receptor-expressing neighboring cells, thus forming syncytia. This powerful bystander effect is important in FMG-mediated antitumor activity; however, expression of the correct cell-surface receptor on the tumor cell is necessary for strong fusogenic activity [33-35]. To determine whether HERV-W 483 could promote cell-cell fusion upon interaction with hASCT2 receptor, HEK293T, HeLa, and TE671 cells were chosen for transfection. As shown in Fig. 3, there was a difference in the degree of syncytium formation in these target cells, which may be due to the different expression levels of the hASCT2 receptor. This is because it is already known that TE671 cells contain both hASCT1 and hASCT2, HEK293T cells express only small amounts of hASCT1 and hASCT2 receptors, and HeLa cells express only hASCT2 receptors [3, 35]. Interestingly, when HEK293T cells were transfected with the full-length, wild-type HERV-W *env* expression plasmid without a 3'-UTR(SPRE), syncytium formation was not observed (Fig. 3A). This result suggests that HERV-W *env* requires a 3'-UTR for efficient gene expression [21, 36]. As previously reported, HERV-W *env* mutant truncated after amino acid 483 induced syncytium formation more efficiently than wild-type HERV-W *env* [4, 19, 20]. Although the insertion of SPRE into HERV-W plasmid induced syncytium formation, HERV-W 483 was more fusogenic than HERV-W-SPRE in HEK293T cells (Fig. 3A). Therefore, HERV-W 483 was chosen for the construction of RCR and s-RCR vectors.

To compare infectivity between the full-length HERV-W *env* and HERV-W 483 mutant, lentiviral vectors pseudotyped with HERV-W *env* were generated (Fig. S1). Consistent with previous results, HERV-W 483 was more efficiently incorporated into pseudotyped viruses than HERV-W *env*, and the titer of HERV-W 483 was higher than that of HERV-W *env* SPRE. Although highly expressed HERV-W 483 induces severe syncytium formation, this envelope protein can still be incorporated into lentiviral vectors to a greater extent than HERV-W *env* SPRE (Figs. 4 and S2). Although it is known that HERV-W *env* cannot be incorporated into MLV particles, Fig. S3 showed that HERV-W *env* can be incorporated into MLV pseudoviruses (Fig. S3). These findings indicate that increasing HERV-W expression by removing the C-terminus or including the intron results in efficient incorporation of HERV-W *env* into pseudoviruses.

To test whether virus-mediated delivery of the HERV-W 483 can induce syncytium formation, we constructed two replication-defective retroviral vectors encoding either HERV-W 483 (*gag-pol* vector; MoMLV-HERV-W 483) or VSV-G (*env* vector; pCLXSN-VSV-G-EGFP). As shown in Fig. 5, s-RCR virus-mediated delivery of the HERV-W 483 induced cell killing through induction of syncytia at day 6. Since s-RCR titers in the supernatants were low due to strong cytotoxicity, these results suggest that s-RCR virus spreads by direct cell-cell contact.

To improve gene delivery, a 10A1-derived RCR vector was used for HERV-W 483 expression (MoMLV-10A1-HERV-W 483). Although MoMLV-10A1-HERV-W 483 contains two types of *env*, the HERV-W 483 Env glycoprotein cannot be incorporated into MLV particles, making this vector exhibit the tropism of 10A1 *env*. 10A1 MLV can infect a wide variety of human cells. It is also known that an RCR vector with a larger insert may be less stable than those with a small insert [13, 22, 23, 28]. Therefore, MLV-derived RCR vectors usually have inserts that are shorter than 1.4 kb. Interestingly, MoMLV-10A1-HERV-W 483-transfected HEK293T cells induced syncytium formation after passage of RCR vector (Fig. 6). This result suggests that our vector containing a 2.1 kb insert (IRES 0.7 kb + HERV-W 483 1.4 kb) is stable. Further studies are needed to test vector instability.

To test RCR viral replication in cancer cell lines, RCR stocks collected from transfected HEK293T cells were used to infect both A549 and HT1080 cells. As shown in Fig. 7, progressively developing toxicity was observed through the RCR vector spreading in these cell lines. Since hASCT2 expression is known to be associated with non-small cell lung cancer [7], the induction of extensive fusion formation in the A549 human lung adenocarcinoma cell line means that hASCT2 is expressed in this cell line. In addition, the RCR vector encoding HERV-W 483 propagated the transgene. This is because the 10A1 envelope from RCR does not interfere with the co-expressed HERV-W *env*. Similar results were reported in previous studies demonstrating that HERV-W *env* could not be incorporated into MLV particles, but HIV-1 could be pseudotyped with the HERV-W *env* [36].

We previously reported that RCR and s-RCR vectors encoding different FMGs are highly promising vectors for cancer gene therapy [16, 37, 38]. Each vector system that encodes a different FMG has advantages and disadvantages. The commonly used GALV FMG is highly cytotoxic and can induce syncytial formation at neutral pH. However, GALV FMG is also highly toxic to virus producer cells, making it difficult to generate high-titer viral vectors. In contrast, VSV-G induces syncytial formation only at low pH and induces an immune response, although it has the advantage of overcoming viral receptor interference. HERV-W FMG, like GALV FMG, has a strong bystander effect and induces a weak immune response because HERV-W *env* is a human antigen. Since HERV-W receptor (hASCT2) is overexpressed in varieties of cancer, the use of HERV-W FMG that selectively binds hASCT2 to form syncytia could be a promising therapeutic strategy. The exact mechanisms of cancer cell death in FMG-induced syncytia are unclear, but multiple pathways, including apoptosis, autophagy, and nonapoptotic cell death, may be involved. Previous studies have shown conflicting results regarding the mechanism of FMG-induced cancer cell death [14, 39-41]. Although apoptosis has been implicated in FMG-induced cancer cell death, it is not the sole mechanism responsible for cancer cell death. For example, Lin *et al*. reported that annexin-V or active caspase-3 staining was not observed in H322 human non-small cell lung cancer (NSCLC) cell lines transfected with HERV-W *env* [13]. This suggests that induction of apoptosis in syncytia is FMG- and cell line-dependent. Moreover, the HERV-W *env* not only has well-known fusion functions, but also non-fusion activities such as apoptosis regulation [42-44]. Further research is needed to elucidate the precise cellular and molecular mechanisms underlying HERV-W *env*-induced syncytial death.

In conclusion, our findings demonstrate that HERV-W 483 exhibits higher fusogenicity compared to the full-length HERV-W *env* when tested in HEK293T cells. This enhanced fusogenicity also leads to syncytium formation in HeLa and TE671 cells. Co-transfection of HEK293T cells with two replication-defective retroviral vectors, one encoding HERV-W 483 and the other encoding VSV-G, results in the progressive development of toxicity. Furthermore, the RCR vector encoding HERV-W 483 remains genetically stable and displays syncytium formation through multiple rounds of replication in human cancer cell lines. Hence, both the s-RCR and RCR vectors encoding HERV-W 483 hold promise as potential agents for FMG-mediated cancer gene therapy.

## Supplemental Materials

Supplementary data for this paper are available on-line only at http://jmb.or.kr.
