# Supplementary material for: A Replication-Competent Retroviral Vector Expressing the HERV-W Envelope Glycoprotein is a Potential Tool for Cancer Gene Therapy
Source: J Microbiol Biotechnol. 2023 Dec 18;34(2):280–8. doi: 10.4014/jmb.2309.09022 (PMC10940750; doi:10.4014/jmb.2309.09022)
Supplement: Supplementary file 1 [file jmb-34-2-280-supple.pdf]

J. Microbiol. Biotechnol.
https://doi.org/10.4014/jmb.2309.09022

Supplementary Figures

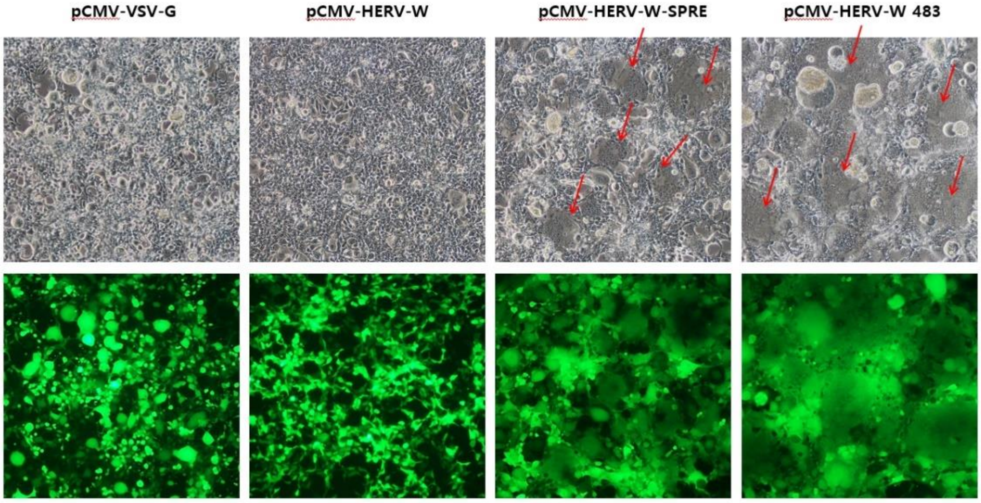

Fig. S1. Generation of HIV-1/HERV-W pseudotyped virus. Lentiviral particles were generated by cotransfection of HEK293T cells with a vector expressing the HERV-envelope (pCMV-HERV-W, pCMV-HERV-W-SPRE, and pCMV-HERV-W 483), pLenti-CMV-GFP-Puro, and psPAX2. pCMV-VSV-G was used as positive control vector. Transfection efficiency of HEK293T cells was determined by fluorescence microscopy at 200 x magnification. Arrows indicate syncytia.

pCMV-VSV-G

Marker	Left, Right	Events	% Gated	% Total	Mean	Geo Mean	CV	Median	Peak Ch
All	1, 9910	10000	100.00	100.00	5995.94	1897.23	70.22	8131.23	9910
M1	30, 9910	8813	88.13	88.13	6801.68	3765.99	56.26	9139.82	9910

pCMV-HERV-W

Marker	Left, Right	Events	% Gated	% Total	Mean	Geo Mean	CV	Median	Peak Ch
All	1, 9910	10000	100.00	100.00	7.23	2.72	752.70	2.46	2
M1	30, 9910	132	1.32	1.32	329.10	176.73	105.23	114.44	938

pCMV-HERV-W-SPRE

Marker	Left, Right	Events	% Gated	% Total	Mean	Geo Mean	CV	Median	Peak Ch
All	1, 9910	10000	100.00	100.00	15.22	2.76	1523.78	2.44	2
M1	30, 9910	167	1.67	1.67	738.87	235.44	222.48	173.09	956

pCMV-HERV-W 483

Marker	Left, Right	Events	% Gated	% Total	Mean	Geo Mean	CV	Median	Peak Ch
All	1, 9910	10000	100.00	100.00	27.34	2.87	1433.70	2.53	2
M1	30, 9910	192	1.92	1.92	1271.24	294.04	199.87	181.87	956

Fig. S2. Determination of HIV-1/HERV-W pseudotype virus infectivity by flow cytometry. To calculate the transduction units per milliliter (TU/mL), the following formula was used.

TU/mL = {(The total number of target cells infected × The frequency of GFP-positive cells determined by flow cytometry / Volume of pseudovirus added (mL)} x Dilution factor.

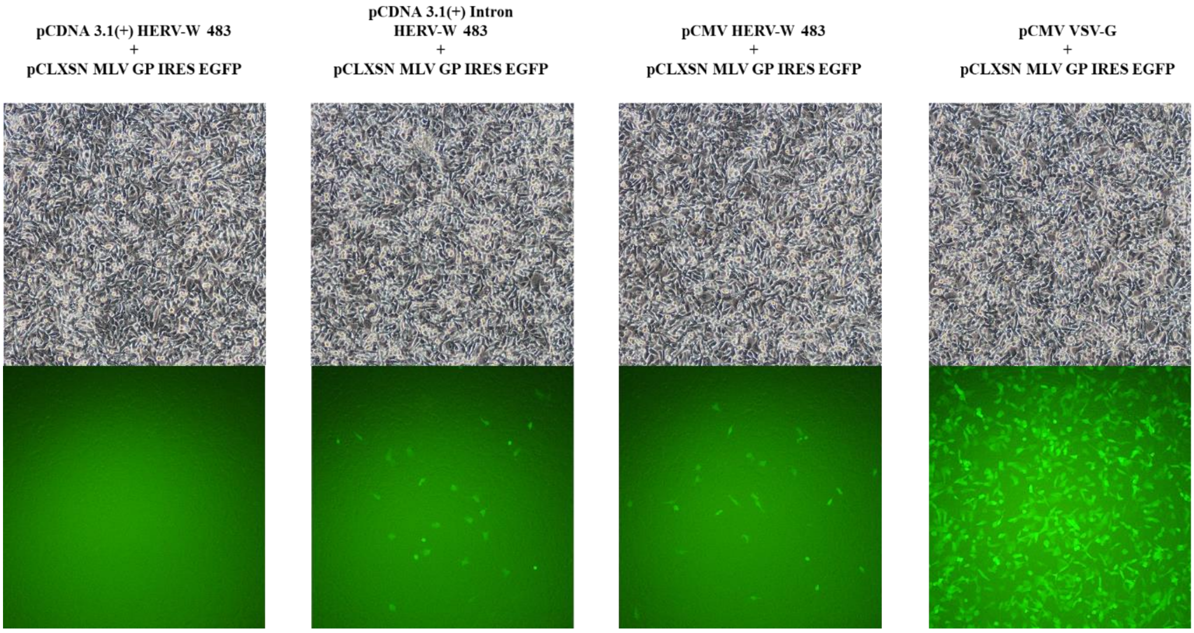

pcDNA 3.1(+) HERV-W 483

Marker	Left, Right	Events	% Gated	% Total	Mean	Geo Mean	CV	Median	Peak Ch
All	1, 9910	10000	100.00	100.00	4.87	4.15	79.19	3.89	3
M1	33, 8977	25	0.25	0.25	46.97	44.36	38.58	38.89	34

pcDNA 3.1(+) Intron HERV-W 483

Marker	Left, Right	Events	% Gated	% Total	Mean	Geo Mean	CV	Median	Peak Ch
All	1, 9910	10000	100.00	100.00	12.30	4.15	889.85	3.79	3
M1	33, 8977	75	0.75	0.75	1035.45	573.37	71.48	1104.00	1512

pCMV HERV-W 483

Marker	Left, Right	Events	% Gated	% Total	Mean	Geo Mean	CV	Median	Peak Ch
All	1, 9910	10000	100.00	100.00	10.61	4.28	909.14	3.89	3
M1	33, 8977	67	0.67	0.67	870.78	379.93	92.69	858.21	39

pCMV VSV-G

Marker	Left, Right	Events	% Gated	% Total	Mean	Geo Mean	CV	Median	Peak Ch
All	1, 9910	10000	100.00	100.00	438.72	26.96	186.05	6.49	4
M1	33, 8977	3165	31.65	31.65	1367.27	1059.63	64.66	1218.81	1197

Fig. S3. Titers of HERV-W 483-pseudotyped retrovirus in HT1080 cells. HEK293T cells were cultured in 6-well plates and co-transfected with 2μg of pCLXSN MLV GP IRES EGFP and 2μg of one of the HERV-W 483-expressing plasmids (pCDNA 3.1(+) HERV-W 483, pCDNA 3.1(+) Intron HERV-W 483, pCMV HERV-W 483, and pCMV VSV-G). The pCMV VSV-G plasmid served as the positive control. HT1080 cells seeded in 6-well plates were infected with 1 ml of pseudotyped viruses, and viral titers were determined by analyzing the proportion of EGFP-positive HT1080 cells using a flow cytometer.
